# Hybrid Organic-Inorganic Materials and Interfaces With Mixed Ionic-Electronic Transport Properties: Advances in Experimental and Theoretical Approaches

**DOI:** 10.3389/fchem.2022.892013

**Published:** 2022-04-12

**Authors:** Mariano Romero, Dominique Mombrú, Fernando Pignanelli, Ricardo Faccio, Alvaro W. Mombrú

**Affiliations:** Centro NanoMat & Área Física, Departamento de Experimentación y Teoría de la Estructura de la Materia y Sus Aplicaciones (DETEMA), Facultad de Química, Universidad de la República, Montevideo, Uruguay

**Keywords:** organic-inorganic hybrid, mixed ionic and electronic conducting, sol-gel—alkoxide route, computational modeling, polymer nano composite

## Abstract

The main goal of this mini-review is to provide an updated state-of-the-art of the hybrid organic-inorganic materials focusing mainly on interface phenomena involving ionic and electronic transport properties. First, we review the most relevant preparation techniques and the structural features of hybrid organic-inorganic materials prepared by solution-phase reaction of inorganic/organic precursor into organic/inorganic hosts and vapor-phase infiltration of the inorganic precursor into organic hosts and molecular layer deposition of organic precursor onto the inorganic surface. Particular emphasis is given to the advances in joint experimental and theoretical studies discussing diverse types of computational simulations for hybrid-organic materials and interfaces. We make a specific revision on the separately ionic, and electronic transport properties of these hybrid organic-inorganic materials focusing mostly on interface phenomena. Finally, we deepen into mixed ionic-electronic transport properties and provide our concluding remarks and give some perspectives about this growing field of research.

## Taxonomy and Definitions

*Hybrid materials* were previously defined by the IUPAC (International Union of Pure and Applied Chemistry) as materials composed of an intimate mixture of inorganic components, organic components, or both types, which usually interpenetrate on scales of less than 1 μm (Alemán et al., 2007). This definition excludes large-sized components for which the term *composite* is usually used in the literature, but does not exclude those named as *nano-composites* (with at least one component exhibiting at least one of its dimensions in the nanoscale) which, in practice, is also considered in the previous *hybrid material* definition. Then, the term *hybrid organic-inorganic (hOI) materials* refer to multi-component compounds having at least one of their organic or inorganic component in the sub-micrometric and, more usually, in the nano-metric size domain (Judeinstein and Sanchez, 1996). *h*OI materials can be classified into two classes named Class I (when organic and inorganic phases interact weakly *via* Van der Waals, hydrogen bonding, or electrostatic interactions) and Class II (when organic and inorganic phases interact strongly *via* chemical bonding interactions), without excluding those interacting simultaneously *via* both types of interactions (Class I and II) (Judeinstein and Sanchez, 1996). Although the previous definitions are more general, in the vast literature *h*OI materials refer mostly to polymers as organic material and metal oxide compounds as inorganic materials. The term *hybrid organic-inorganic polymers* has also been used in the literature mostly referring to organic polymers and inorganic materials blended by mutual dispersion at molecular dimensions (Schmidt, 1992; Saegusa, 1995). This terminology can be useful as it excludes the inorganic materials in the form of inorganic particles or nanoparticles and refers mostly to inorganic clusters on the molecular or macromolecular scale. However, other authors simply refer to the laters by using the *h*OI materials terminology as it still fits perfectly to its definition which does not specify a lower limit for inorganic components size (Sanchez and In, 1992; Sanchez and Ribot, 1993; Judeinstein and Sanchez, 1996). Our major focus in this mini-review will be given to organic-inorganic interface phenomena for *h*OI materials belonging to both Class I and Class II but only those involving organic polymers and metal oxide nanomaterials.

## Preparation, Characterization and Computational Simulation of *h*OI Materials and Interfaces

### Solution-Phase Reaction of Inorganic/Organic Precursor Using Organic/Inorganic Hosts

**FIGURE 1:**
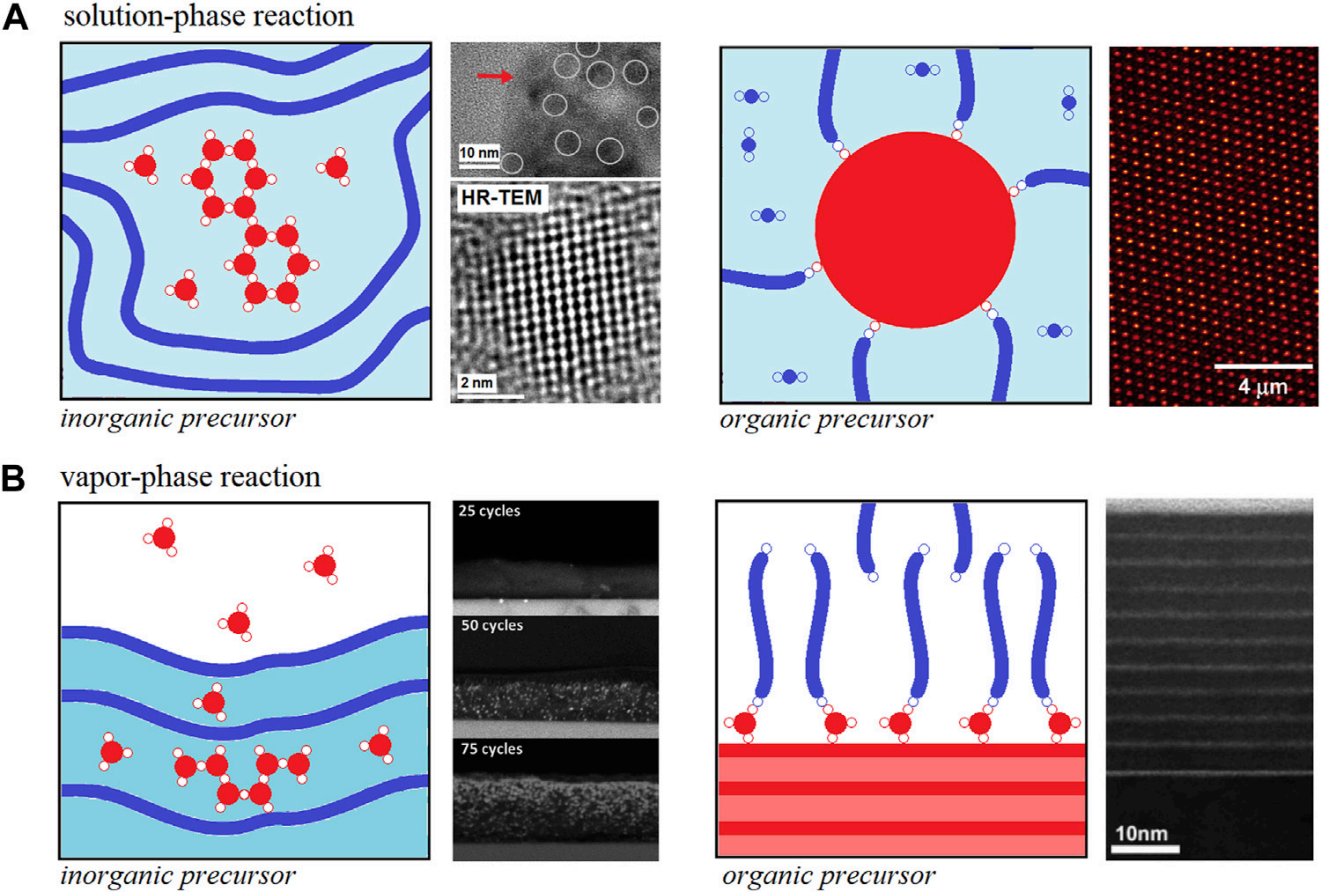
Schematic representation for **(A)** solution-phase and **(B)** vapor phase reactions to obtain *h*OI materials. Both cases include the preparation of *h*OI materials at expenses of inorganic precursors growth in the presence of organic polymer host (left panels) and using organic precursors polymerization in the presence of inorganic clusters/nanoparticles or surfaces (right panels). Each panel also includes: HRTEM images for PANI-TiO_2_ prepared via solution-phase reaction of titanium propoxide and polyaniline with further exposure to water vapor (A, left panel) [*Portions of figures adapted/altered minimally with permission from Elsevier*] (Mombrú et al., 2017a), confocal laser scanning microscopic images for PMMA-SiO_2_ prepared via surface-initiated atom transfer radical polymerization of MMA (A, right panel) [*Portion of figure adapted/altered minimally with permission from American Chemical Society*] (Ohno et al., 2006), Cross-section HRSEM images for P3HT-ZnO films obtained after 25, 50, 75 diethylzinc/water ALD cycles (B, left panel) [*Portions of figure adapted/altered minimally with permission from Royal Society of Chemistry*] (Obuchovsky et al., 2014). Cross-section HRTEM images of self-assembled organic multilayer/TiO_2_ nanolaminate films made by using sequential ALD/MLD cycles of titanium isopropoxide and 7-octenyltrichlorosilane vapors, respectively (B, right panel) [*Portion of figure adapted/altered minimally with permission from American Chemical Society*] (Lee et al., 2007).

The solution-phase reaction of inorganic precursors into organic hosts such as organic polymers was first reported in the early nineties (Sanchez and In, 1992; Schmidt, 1992; Sanchez and Ribot, 1993; Saegusa, 1995). The mechanism for metal alkoxides hydrolysis-condensation towards metal oxide clusters or particles (widely known as the sol-gel method) has been thoroughly studied since the late sixties and it is quite well-understood (Livage et al., 1988; Brinker and Scherrer, 1989). Furthermore, the solution-phase reaction of inorganic precursors (metal alkyl or alkoxide) in the presence of organic polymers essentially follows the ordinary hydrolysis-condensation mechanism. However, the presence of certain functional groups in the polymer structure can interact *via* non-chemical (Class I) and chemical (Class II) bonding interactions with the metal alkoxide hydrolysis-condensation products at different stages such as the case depicted in Figure 1A—left panel.

On the other hand, the solution-phase reaction of organic precursor (organic monomer or oligomer) in the presence of inorganic hosts is a quite different process as the organic polymerization is the main reaction taking place. In this case, the inorganic hosts are usually nanoparticles or well-defined clusters interacting *via* non-chemical interactions during the organic polymerization (Class I) but these nanoparticles or well-defined clusters can be also pre- or post-functionalized with the organic monomer or oligomer to act as an initiator for organic polymerization (Class II) such as the case depicted in Figure 1A—right panel (Ohno et al., 2006). The mechanism behind *h*OI materials preparation is much more complex and will not be discussed further in this mini-review but the reader can refer to some outstanding reviews on this topic in the literature (Saegusa, 1995; Judeinstein and Sanchez, 1996; Sanchez et al., 2001; Krasia-Christoforou, 2015; Zhang et al., 2016a; Gon et al., 2017).

### Vapor-Phase Infiltration of the Inorganic Precursor Into Organic Hosts and Molecular Layer Deposition of Organic Precursor Onto the Inorganic Surface

Over the past decade, a new approach to obtain *h*OI materials has emerged in which organic polymers are exposed to metalorganic vapors using different approaches such as *multiple pulsed infiltration*, (Lee et al., 2009; Lee et al., 2010), *sequential infiltration synthesis* (Peng et al., 2011; Tseng et al., 2011; Segal-Peretz et al., 2015) and *sequential vapor infiltration* (Gong et al., 2011; Dandley et al., 2014; Akyildiz et al., 2014; Obuchovsky et al., 2014). These approaches essentially respond to the same general mechanism involving the diffusion of inorganic precursor (metal alkyl or alkoxide) molecules into a “dried” organic polymer yielding to Class I and Class II *h*OI materials and, thus, the unifying term *vapor-phase infiltration* (VPI) processes and its corresponding reaction mechanisms have been recently proposed to describe all of them (Leng and Losego, 2017). VPI into different kinds of organic polymers [*e.g.* silk, collagen, polymethyl methacrylate (PMMA), polystyrene (PS), polybutylene terephthalate (PBT), polyethylene terephthalate (PET), polylactic acid (PLA) and polyethylene naphthalate (PEN)] using trimethylaluminum, diethylzinc and titanium isopropoxide precursors have proven to be successful routes toward hybrid polymers with Al_2_O_3_, TiO_2_ and ZnO interesting nanostructures such as the case depicted in Figure 1B—left panel (Lee et al., 2009; Lee et al., 2010; Gong et al., 2011; Peng et al., 2011; Tseng et al., 2011; Akyildiz et al., 2014; Dandley et al., 2014; Obuchovsky et al., 2014; Segal-Peretz et al., 2015; Leng and Losego, 2017).

On the other hand, the vapor-phase infiltration of organic precursors (organic monomer or oligomer) into “dried” inorganic hosts is not referred to as such in the literature as it is mostly limited to surface phenomena and the term *molecular layer deposition* (MLD) of organic precursor onto the inorganic surface is defined instead (Gregorczyk and Knez, 2016; Meng, 2017). MLD of organic precursors onto inorganic surfaces is a sister of the *atomic layer deposition* (ALD) technique but still much less explored due to the complexity of its mechanism (Gregorczyk and Knez, 2016; Meng, 2017). However, the successful advances using MLD for obtaining nanoscale films of organic polymers (Yoshimura et al., 1991; Yoshimura et al., 1992; Kim et al., 2005; Zhou et al., 2013; Atanasov et al., 2014; Kim et al., 2015) have also encouraged the preparation of *h*OI materials such as metal-based hybrid polymers named as alucones, zincones, titanicones among others (Dameron et al., 2008; Lee et al., 2007; Peng et al., 2009; Abdulagatov et al., 2013; Yoon et al., 2012; Park et al., 2016; Van de Kerckhove et al., 2016; Nisula and Karppinen, 2016). In the latter case, the host inorganic surface reacts with the metal-organic precursor vapor and once it is grafted to the surface it allows further organic oligomerization processes and, that is probably why most cases refer to Class II *h*OI materials such as the case depicted in Figure 1B—right panel (Yoshimura et al., 1991; Yoshimura et al., 1992; Kim et al., 2005; Lee et al., 2007; Dameron et al., 2008; Peng et al., 2009; Yoon et al., 2012; Abdulagatov et al., 2013; Zhou et al., 2013; Atanasov et al., 2014; Kim et al., 2015; Nisula and Karppinen, 2016; Park et al., 2016; Van de Kerckhove et al., 2016; Meng, 2017).

### Computational Simulation Insights on Structural Features of *h*OI Materials and Interfaces

Computational simulation is quite relevant to predict or explain structural features of *h*OI materials and interfaces. For instance, classical Montecarlo (MC) and molecular dynamics (MD) are one of the best (non-atomistic and atomistic, respectively) approaches to model up to several nanometer large crystalline or amorphous organic and inorganic materials allowing obtaining rich information about molecular and nuclear position-related properties (Zeng et al., 2003; Heinz and Ramezani-Dakhel, 2016; Ramakrishnan et al., 2017; Eckert et al., 2020). To compute electronic-related properties, we must perform first-principles calculations such as Density Functional Theory (DFT) and ab-initio MD (AIMD) to access static and dynamic information, respectively (Zeng et al., 2003; Heinz and Ramezani-Dakhel, 2016; Ramakrishnan et al., 2017; Eckert et al., 2020). When performing first-principles calculations, due to the complexity of the *h*OI interface, one of the organic or inorganic phases is usually oversimplified to systems comprising organic monomer/inorganic surface (Alexandre et al., 2010; Semoto et al., 2011; Hofmann et al., 2013; Motta et al., 2015; Wang et al., 2017a; Pourrahimi et al., 2017; Liao et al., 2019) or organic oligomers/inorganic small clusters (Mombrú et al., 2017a; Ullah et al., 2017; Wang et al., 2020a). The rational simplification of the *h*OI interface model is a key to computing these calculations using an appropriate level of theory at a reasonable computational cost (Hofmann et al., 2021).

There are a lot of experimental characterization techniques to study *h*OI materials and interfaces such as nuclear magnetic resonance, X-ray photoemission, vibrational and optical spectroscopies to access chemical features and high-resolution electron, atomic force and tunneling microscopies, in addition to small/wide-angle X-ray scattering, to access structural features of *h*OI interfaces. Probably because of their popularity, infrared and Raman spectroscopies are the most commonly observed in the literature to study the organic-inorganic interactions mainly for Class II *h*OI materials (Atanasov et al., 2014; Yoon et al., 2012; Mombrú et al., 2017a; Wang et al., 2020a; Schöttner et al., 2020). Moreover, surface-enhanced Raman scattering (SERS) effect without using noble metals but inorganic (*e.g.* TiO_2_, MoO_3_, WO_3_, NiO, ZnO and Cu_2_O) (Sun et al., 2007; Alessandri, 2013; Bontempi et al., 2014; Qi et al., 2014; Shin et al., 2014; Wang et al., 2014; Cong et al., 2015; Bontempi et al., 2016; Wang et al., 2017b; Lin et al., 2017; Wu et al., 2017; Zhang et al., 2017; Demirel et al., 2018) or organic (*e.g.* graphene, thiophene oligomers) (Ling and Zhang, 2010; Yu et al., 2011; Ling et al., 2012; Ling et al., 2013; Huang et al., 2015; Kang et al., 2015; Zhang et al., 2016b; Lombardi, 2017; Yilmaz et al., 2017; Liu et al., 2018) semiconductors is rising and appears to be a very promising technique to study *h*OI interfaces. Not only there is a vast experimental database that can be useful for vibrational modes assignment but also infrared and, to a lesser extent, Raman spectra can be easily computed using first-principles calculations (Mombrú et al., 2017a; Wang et al., 2020a; Schöttner et al., 2020; Fernández-Werner et al., 2017; Pignanelli et al., 2017). Undoubtedly, nuclear magnetic resonance and X-ray photoelectron spectroscopies are other powerful tools to obtain chemical information about *h*OI interfaces but are less accessible than vibrational spectroscopies and computational calculations demand a high computational cost. High-resolution electron, atomic force and tunneling microscopies can give direct local information about *h*OI materials and interfaces but it is almost impossible to access a large amount of sample as in the case of using X-ray scattering techniques, thus both techniques should be complemented. Furthermore, the modeling of X-ray scattering data by using Rietveld (for crystalline) or Debye (for both crystalline and amorphous) methodologies are quite simple to perform and can be also assisted by classical molecular dynamics calculations (Scardi and Gelisio, 2016; Fernández-Werner et al., 2017; Pignanelli et al., 2017; Bokuniaeva and Vorokh, 2019; Bertolotti et al., 2020).

## Mixed Ionic-Electronic Transport of *h*OI Materials and Interfaces

### Ionic Transport

**FIGURE 2:**
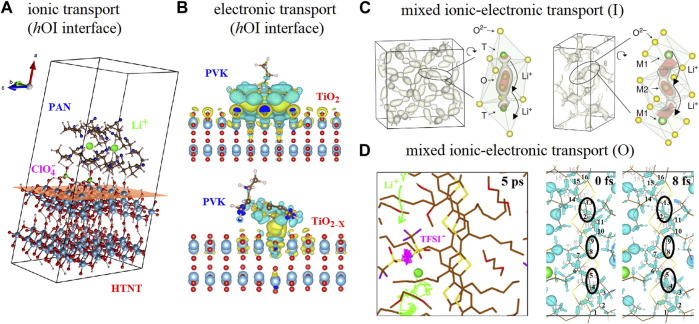
**(A)** AIMD calculations showing the Li^+^ - ClO_4_^−^ ionic-pair dissociation for an *h*OI interface based on polyacrylonitrile (PAN) and titanate surface (HTNT) [*Portion of figure adapted/altered minimally with permission from American Chemical Society*] (Pignanelli et al., 2018a). **(B)** DFT calculations showing the electronic charge transfer interactions between vinyl carbazole monomer (PVK) and TiO_2_ surface including oxygen vacancies (TiO_2-x_) [*Portion of figure adapted/altered minimally with permission from Elsevier*] (Mombrú et al., 2019b). **(C)** AIMD calculations showing the probability density of Li^+^ spatial occupancy and the elongation feature of probability density along the migration channel with the corresponding isosurfaces for LLZO and LATP [*Portion of figure adapted/altered minimally with permission from Nature Publishing Group*] (He et al., 2017). **(D)** AIMD calculations showing Li^+^ and TFSI^−^ transport at the P3HT side chain region mediated by glycol molecules (t < 5 ps) and the electron-phonon mediated electronic transport at the P3HT backbone (t < 20 fs) [*Portion of figure adapted/altered minimally with permission from American Chemical Society*] (Mombrú et al., 2022).

Since the early nineties, it has been evidenced that the presence of inorganic nanomaterials into polymer matrices increases the ionic conductivity and improves the mechanical properties of solid polymer electrolytes (Croce et al., 1998; Appetecchi et al., 1998; Croce et al., 2000; Serra Moreno et al., 2014). Although most of these cases involve no chemical bonding between inorganic nanomaterials and polymers, there is a vast variety of inorganic nanomaterials morphologies such as nanoparticles, nanorods and nanotubes acting as both passive and active fillers that have yielded an enhancement on the ionic conductivities (Romero et al., 2015; Liu et al., 2015; Pignanelli et al., 2018a; Pignanelli et al., 2018b). The enhancement of ionic conduction due to the presence of inorganic nanostructures is mostly related to the ionic-pair dissociation of ordinary salts mediated by the interaction with the inorganic surface, thus favoring the fixation of anionic species and favoring the cationic conduction as evidenced by Raman microscopy and schematically depicted in Figure 2A (Romero et al., 2015; Pignanelli et al., 2018a; Pignanelli et al., 2018b). There are other *h*OI materials involving chemical bonding between organic and inorganic oligomers that have shown an enhancement in the ionic conductivity with respect to their organic counterparts (Saikia et al., 2012; Vélez et al., 2013; Saikia et al., 2014). Other approaches using borate and carbonate salts as precursors where the “undesired” mobile anionic species are eliminated or chemically bonded to the polymer have been also reported favoring single ionic conduction of the “desired” mobile cation (Zhu et al., 2013; Zhang et al., 2014; Pignanelli et al., 2019). However, the advances on single lithium-ion conductors based on Class II *h*OI materials comprising well-defined inorganic clusters chemically bonded to organic polymers represent a more rational and promising approach (Onishi et al., 1996; Fujinami et al., 1997; Onishi et al., 1998; Fujinami et al., 2000; Villaluenga et al., 2013; Meyer et al., 2014; Zhao et al., 2015a; Zhao et al., 2015b; Lago et al., 2015).

Regarding computational simulation studies, there is a relatively easy and low-cost DFT approach that provides insights on the interactions between ions and organic molecules/inorganic surfaces that can be used to explain dissociation processes but only represents a static picture without giving direct information about transport (Romero et al., 2016; Kwon et al., 2018; Pignanelli et al., 2019; Xie et al., 2019). To have direct insight on ionic transport for *h*OI materials and interfaces, larger-scale approaches using non-atomistic or atomistic calculations including organic oligomers and inorganic clusters or surfaces are necessary. MD can provide rich information about ion diffusion and transport directly from the trajectories but these calculations need to be conducted over sufficiently long time scales (Webb et al., 2015; Deng et al., 2016; Sun et al., 2016; Mogurampelly et al., 2017; Pignanelli et al., 2017; Xue et al., 2017; Patra et al., 2019; Pignanelli et al., 2021). However, such long-time scales are highly demanding for AIMD and this first-principle approach is rare in large systems (such as amorphous solids) and it is more usually performed for relatively small crystalline systems (Jalem et al., 2013; Meier et al., 2014; Mo et al., 2014). Furthermore, other first-principles calculations based on the nudged elastic band (NEB) method are also commonly used to determine the barrier of the ionic migration but it is also particularly useful for inorganic crystalline systems (Ong et al., 2011; Shi et al., 2012; Mo et al., 2014; Bachman et al., 2015; Moriwake et al., 2015; Zhang et al., 2018). Nonetheless, the vast majority of lithium-ion transport calculations are solely within the bulk of organic (Webb et al., 2015; Sun et al., 2016; Mogurampelly et al., 2017; Pignanelli et al., 2017; Xue et al., 2017; Patra et al., 2019; Pignanelli et al., 2021) or inorganic (Ong et al., 2011; Shi et al., 2012; Mo et al., 2014; Bachman et al., 2015; Moriwake et al., 2015; Deng et al., 2016; Zhang et al., 2018) phases, but still rarely for *h*OI materials and interfaces (Li et al., 2016; Pignanelli et al., 2018a; Li et al., 20202020). Recently, there have been interesting approaches based on AIMD and NEB calculations studying the solid electrolyte interface (SEI) comprising organic oligomers and metallic lithium electrodes (Yildirim et al., 2017; Merinov et al., 2019; Ramasubramanian et al., 2019; Merinov et al., 2020).

### Electronic Transport

The electronic interactions in *h*OI materials have been extensively studied particularly for sensing, energy storage and energy conversion applications (Kumar et al., 2018; Singh et al., 2019; Duan et al., 2020; Niederhausen et al., 2021; Rathnayake et al., 2021; Zhang et al., 2021). The electronic interactions in these *h*OI materials and interfaces are mostly *via* proximity of the *π*-cloud of the organic phase with the inorganic surface, and thus Class I *h*OI materials are the most studied for these purposes. However, there have recent advances in Class I and II *h*OI materials comprising well-defined inorganic clusters with promising properties in a broad range of applications (Horn et al., 2021). One of the major challenges of *h*OI materials for polymer solar cells applications is the substitution of the fullerene electronic acceptors for inorganic metal oxides nanostructures counterparts in the active layer and also the enhancement of electronic transport at the *h*OI interface in the electrodes (Moet et al., 2007; Bouclé et al., 2008; Dridi et al., 2008; Lin et al., 2009; Chandrasekaran et al., 2011; Aashish et al., 2016; Ikram et al., 2016). The later reports comprise *h*OI materials based on different electronic conducting polymers with ZnO, TiO_2_ and In-doped SnO_2_ (ITO) inorganic nanostructures or surfaces (Moet et al., 2007; Bouclé et al., 2008; Dridi et al., 2008; Lin et al., 2009; Chandrasekaran et al., 2011; Aashish et al., 2016; Ikram et al., 2016). However, only a few studies have provided more fundamental insights on the electronic interactions and correlations with structural features for *h*OI materials and interfaces (Schlesinger et al., 2013; Whittaker-Brooks et al., 2014; Mombrú et al., 2017b; Mombrú et al., 2018; Singh et al., 2019).

As we mentioned earlier, it is quite challenging to perform computational simulation of *h*OI interfaces using first-principles level of theory and most calculations usually refer to organic molecules or oligomers interacting with inorganic surfaces. For instance, DFT studies on the electronic interactions of tetrafluoro-tetracyanoquinodimethane (F4-TCNQ)/ZnO surfaces (Xu et al., 2013), hexafluoro-tetracyano-naphthoquinodimethane (F6-TCNNQ)/ZnO surfaces (Schöttner et al., 2020), oligothiophenes and ZnO (and In-doped SnO_2_) surfaces (Timpel et al., 2020) has shed some light on the work function and other relevant electronic properties of these *h*OI interfaces. Other DFT studies on the electronic interactions between larger and more complex systems such as polyaniline oligomer/graphene oxide quantum dots (GQD) including edge functionalization (Mombrú et al., 2017c) and vinyl carbazole monomer/TiO_2_ surfaces including oxygen and titanium vacancy defects (Mombrú et al., 2019a; Mombrú et al., 2019b) has shown interesting features on the electronic properties evidencing the relevance of using more realistic models as depicted in Figure 2B. For instance, the difference between the LUMO level of vinyl carbazole and the conduction band of TiO_2_ surface with oxygen vacancies decreases thus favoring the energy barrier associated with the charge injection at this *h*OI interface (Mombrú et al., 2019b).

### Mixed Ionic-Electronic Transport

There are a lot of recent examples of *h*OI materials with promising mixed ionic-electronic transport properties in different applications such as lithium and sodium-ion battery electrode (Sengodu and Deshmukh, 2015) and bio-electronic materials (Kousseff et al., 2022; Kukhta et al., 2022). There are also recent reviews on the most relevant and popular techniques that can be useful to characterize experimentally the mixed ionic-electronic transport in *h*OI materials (Romero et al., 2020; Wu et al., 2022). However, there are still very few reports dealing with computational simulation approaches of mixed ionic-electronic transport of *h*OI materials and interfaces mainly due to their extremely demanding computational cost. In the last years, there have been some advances but only for isolated inorganic (Nakayama et al., 2012; Suthirakun et al., 2014; He et al., 2017; Taylor et al., 2017; Kim et al., 20182019; Parras et al., 2018; Wind et al., 2018; Griffith et al., 2019; Heifets et al., 2019; Kang et al., 20192019; Wang et al., 2020b) and organic (Dong et al., 2019; Matta et al., 2020; Mombrú et al., 2020; Dong et al., 2021; Onorato et al., 2021; Zozoulenko et al., 2021; Mombrú et al., 2022) phases revealing quite interesting features.

For inorganic materials, the mixed ionic-electronic transport has been thoroughly studied for solid oxide combustible cells electrode materials (Sunarso et al., 2008) and DFT calculations have recently evidenced the simultaneous positional rearrangement of localized electrons during the oxygen vacancy ionic jump process following a concerted mechanism (Nakayama et al., 2012; Taylor et al., 2017). However, experimental and computational simulations on the mixed ionic-electronic transport in inorganic materials based on protons, lithium, sodium and potassium ions have been explored just recently (He et al., 2017; Wind et al., 2018; Griffith et al., 2019; Kang et al., 20192019; Wang et al., 2020b). Analyzing Li + dynamics from AIMD simulations, He *et al* have evidenced that most Li ions migrate in a highly concerted fashion which means that multiple ions hop simultaneously into their nearest sites within a few picoseconds as depicted in Figure 2C (He et al., 2017). Griffith *et al* have just evidenced by DFT calculations that, Li^+^ in TiNb_2_O_7_ with high states of lithiation exhibits a transition from interstitial-mediated to a vacancy-mediated diffusion mechanism, and the vacancy formation energetics may become rate-determining (Griffith et al., 2019). Within the same framework, the diffusion of Na^+^, K^+^, and Mg^2+^ was also examined in these inorganic structures and these cations exhibit very high diffusion barriers suggesting minimal ionic conduction at room temperature (Griffith et al., 2019). However, in the later report authors remark that they have used standard DFT to capture the mechanisms associated with ionic diffusion without the presence of simultaneous additional electron transfer processes and that it would be also important to further consider the full compositional range, not only the host structure and/or end point (Griffith et al., 2019).

For organic materials, due to their typical amorphous and/or crystalline nature, larger systems including the evaluation of these structural features are usually studied by MD calculations, at least to evaluate their effects on the isolated ionic or (indirectly) electronic transport (Dong et al., 2019; Mombrú et al., 2020; Dong et al., 2021; Onorato et al., 2021). Furthermore, MD simulations have been useful to evidence that, upon swelling cations interact with the polymer side chains while upon doping the excess anions penetrating the polymer microstructure are expected to be more closely interacting with the polymer backbone, stabilizing polarons (Matta et al., 2020; Cendra et al., 2019; Flagg et al., 2018; Inal et al., 2016; Savva et al., 2019). A quite challenging simultaneous access to ionic and electronic transport by AIMD calculations has been reported just recently for a system comprising crystalline P3HT polymer using explicit LiTFSI dopant and glycol molecules depicted in Figure 2D (Mombrú et al., 2022). In the later report, both ionic and electronic transport simultaneous calculations showed a good correlation with the experimental reports of similar mixed ionic−electronic conductors. Furthermore, these AIMD calculations have also allowed introducing the role of the explicit dopant in the interchain, intrachain, “effective” doping, and charge-transfer complex bonding distances, and their associated static and dynamic disorder effects on electronic transport (Mombrú et al., 2022).

## Final Remarks and Perspectives

To make some final comments and define some perspectives, we conclude that, although quite computationally expensive, AIMD seems to be the best choice for modeling *h*OI interfaces as the mixed ionic-electronic transport of both organic and inorganic phases can be adequately modeled simultaneously and be compared with the available literature on their isolated counterparts. However, only a few of the computational simulation from those described above comprises mixed ionic-electronic transport using AIMD calculations (He et al., 2017; Taylor et al., 2017; Wind et al., 2018; Mombrú et al., 2022) so more efforts are definitively still needed in this field. It is also important to mention that the mixed ionic-electronic transport of *h*OI materials is much more complex as it includes crystalline and amorphous regions well above the nm scale that be no longer accessed through atomistic models. Furthermore, relevant processes in experimental devices occurring on the μm scale are out of reach for quantum chemistry and molecular dynamics, and thus continuum models also play an important role here to provide understanding at this level (Zozoulenko et al., 2021).
